# How the Destabilization of a Reaction Intermediate Affects Enzymatic Efficiency: The Case of Human Transketolase

**DOI:** 10.1021/acscatal.9b04690

**Published:** 2020-02-07

**Authors:** Mario Prejanò, Fabiola E. Medina, Maria J. Ramos, Nino Russo, Pedro A. Fernandes, Tiziana Marino

**Affiliations:** † Dipartimento di Chimica e Tecnologie Chimiche, Università della Calabria, 87036 Arcavacata di Rende (CS), Italy; ‡ UCIBIO, REQUIMTE, Departamento de Química e Bioquímica, Faculdade de Ciências, Universidade do Porto, Rua do Campo Alegre s/n, 4169-007 Porto, Portugal

**Keywords:** transketolase, adduct, distortion, QM/MM, DFT

## Abstract

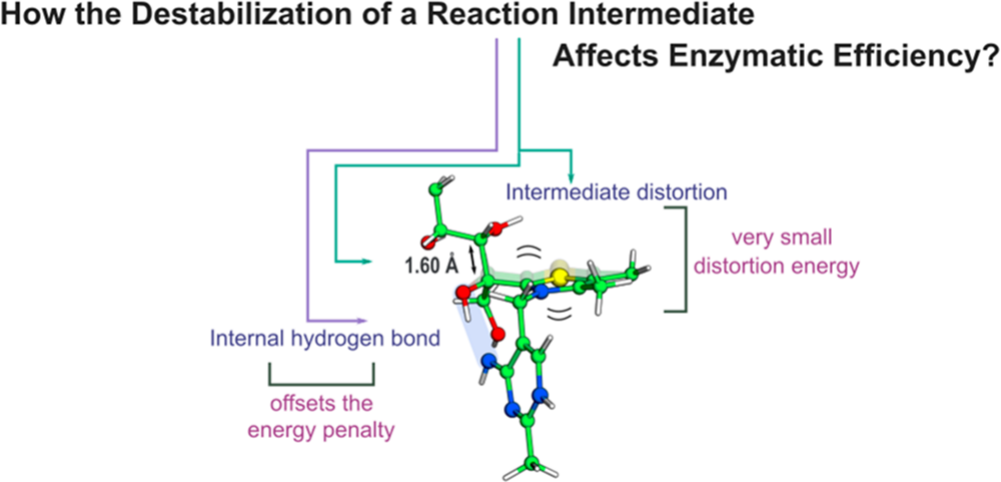

Atomic resolution X-ray crystallography has shown that an intermediate (the X5P-ThDP adduct) of the catalytic cycle of transketolase (TK) displays a significant, putatively highly energetic, out-of-plane distortion in a *sp*^*2*^ carbon adjacent to a lytic bond, suggested to lower the barrier of the subsequent step, and thus was postulated to embody a clear-cut demonstration of the *intermediate destabilization* effect. The lytic bond of the subsequent rate-limiting step was very elongated in the X-ray structure (1.61 Å), which was proposed to be a consequence of the out-of-plane distortion. Here we use high-level QM and QM/MM calculations to study the *intermediate destabilization* effect. We show that the intrinsic energy penalty for the observed distortion is small (0.2 kcal·mol^–1^) and that the establishment of a favorable hydrogen bond within X5P-ThDP, instead of enzyme steric strain, was found to be the main cause for the distortion. As the net energetic effect of the distortion is small, the establishment of the internal hydrogen bond (−0.6 kcal·mol^–1^) offsets the associated penalty. This makes the distorted structure more stable than the nondistorted one. Even though the energy contributions determined here are close to the accuracy of the computational methods in estimating penalties for geometric distortions, our data show that the *intermediate destabilization* effect provides a small contribution to the observed reaction rate and does not represent a catalytic effect that justifies the many orders of magnitude which enzymes accelerate reaction rates. The results help to understand the intrinsic enzymatic machinery behind enzyme’s amazing proficiency.

## 1 Introduction

Enzymes play an essential role in a broad variety of biochemical processes. Understanding these processes is an interest, and a major challenge, for the research community.^1,2^ The most popular theory about the origin of enzyme’s catalytic power was proposed by Pauling in 1948.^3^ The underlying idea is that enzymes catalyze reactions by binding better the transition state than the ground state, which is materialized through a higher binding affinity for the former. Several proposals have been put forward to explain the physical origin of the enzyme transition state stabilization, in particular to show why and how this stabilization is significantly larger than transition state stabilization provided by the solvent in the corresponding uncatalyzed aqueous solution reaction.^4−14^

One of these proposals is based in *ground-state destabilization*. The proposal suggests that the enzyme rate constant (*k*_cat_), and thus the enzyme’s efficiency (*k*_cat_/*K*_M_), is very dependent on the substrate conformation in the Michaelis complex.^14,15^ A resulting aspect is that a substrate that binds the enzyme in a conformation that looks like the transition state (which can be seen as “distorted” when compared to the lower-energy aqueous solution conformation) needs to climb a lower barrier to reach the transition state, thus increasing *k*_cat_.^16−18^ This very interesting proposal is not free from controversy, as the *k*_cat_ increase might be achieved at the cost of increasing *K*_M_ as well, and thus it is not clear how the *ground-state destabilization* does increase the enzyme efficiency (*k*_cat_/*K*_M_), which is the relevant rate constant in physiologic conditions.^13^ The case of the *intermediate destabilization* is different, however, as the substrate still binds the enzyme in the relaxed conformation (without increasing *K*_M_) but is “protected” from falling into low-energy intermediates (through enzyme-induced distortion) that would trap it in the bottom of high-barrier wells. In this sense, the *intermediate destabilization* might indeed increase *k*_cat_ without increasing *K*_M_.

**Scheme 1:**
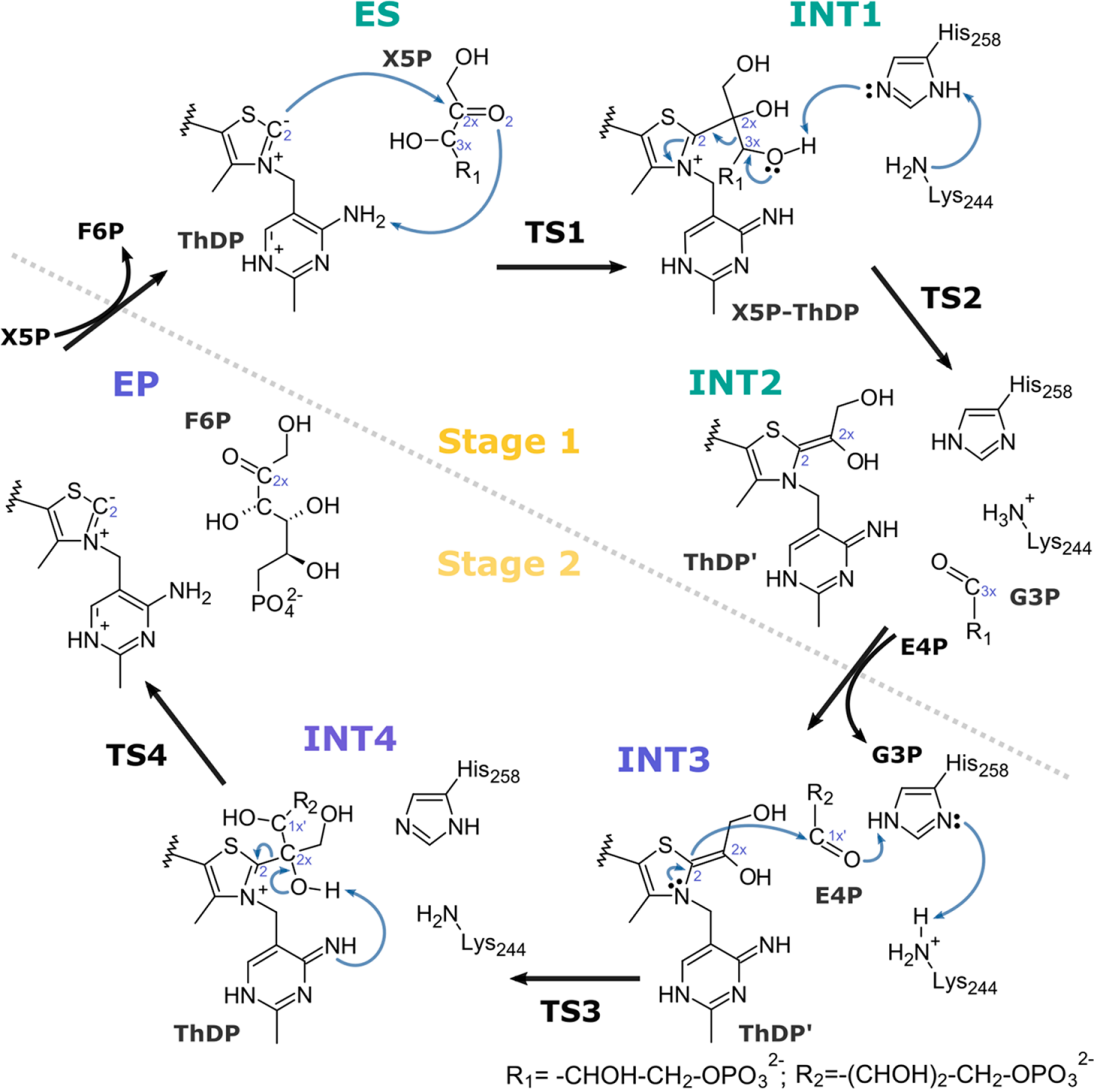
Catalytic Mechanism of the Human Transketolase

Human transketolase (hTK) is a thiamine diphosphate (ThDP)-dependent enzyme that catalyzes a two step reaction consisting in the transfer of a dihydroxyethyl group from the ketose d-xylulose-5-phosphate (X5P) to the aldose d-erythrose-4-phosphate (E4P), to yield the products d-fructose-6-phosphate (F6P) and d-glyceraldehyde-3-phosphate (G3P). We have recently studied the catalytic mechanism of TK with a cluster model approach, and the uncovered mechanism is shown in Scheme 1.^19^

**Figure 1:**
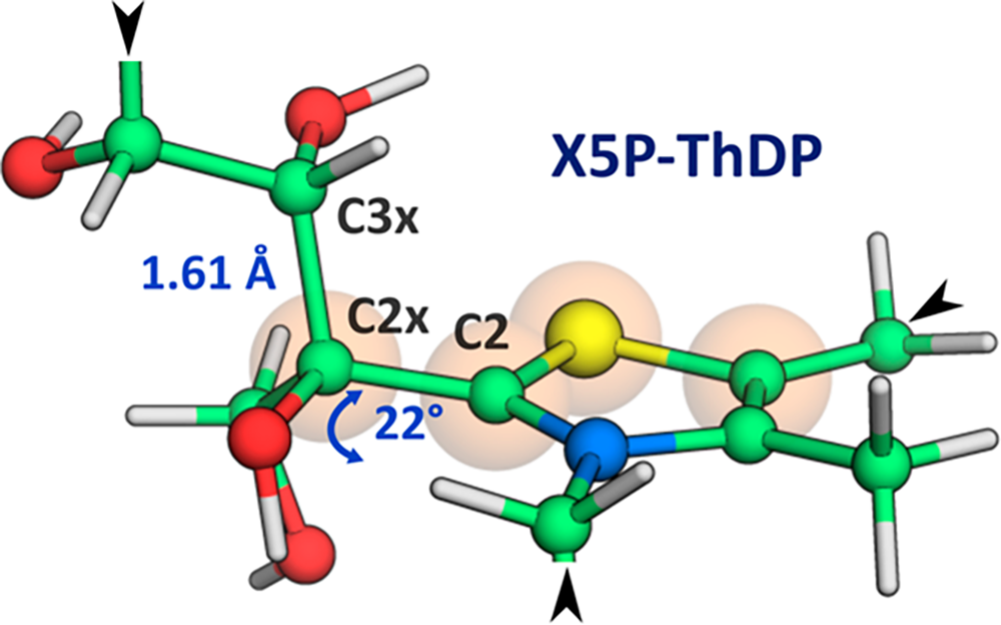
Covalent X5P-ThDP adduct in the high-resolution hTK X-ray structure 4KXV. The atoms highlighted mark the dihedral angle whose distortion brings C2x out-of-plane. The bond to be broken in the subsequent step involves the C2x and the C3x atoms. The black triangles pointing to the atoms represent the rest of the adduct X5P-ThDP, not represented in the figure for simplicity.

The investigated mechanism proposes two transition states interlinked by a stable intermediate, for each stage of the entire catalytic cycle, in which the formation of the products is accelerated by the catalytic dyad His-Lys, lying in the active site.^18^ A high-resolution (0.97 Å) crystallographic structure of hTK recently reported^20^ has revealed an out-of-plane distortion in one of its key intermediates, namely the X5P-ThDP adduct (**INT1** in Scheme 1). This species exhibits an out-of-plane deviation of 22° in relation to the ideal C2-atom *sp*^*2*^ planar geometry,^17,20−22^ as shown in Figure 1. It was proposed that the distortion raises the energy of **INT1** and thus lowers the barrier of the subsequent step, contributing to increase the enzyme’s *k*_*cat*_. This is an attractive hypothesis, in particular because we have shown before that **INT1** is one of the lowest-energy minima in the overall catalytic cycle (2.3 kcal·mol^–1^ above the absolute minimum) and thus can easily become a rate-determining state if it is allowed to relax further down. Furthermore, the X5P-ThDP intermediate exhibits what was considered to be a highly strained, elongated, C2x-C3x scissile bond (1.61 Å), the bond that will be cleaved in the subsequent reaction step.^22^ These experimental pieces of evidence point to an *intermediate destabilization* catalytic effect.

Stimulated by this unusual case, we have studied the effect of the X5P-ThDP out-of-plane distortion in the reaction rate with a complete enzyme model described at the hybrid quantum mechanical/molecular mechanical (QM/MM) level, to evaluate if the out-of-plane distortion translated into a relevant catalytic *intermediate destabilization* effect. Additionally, we wanted to understand the possible relationship between the X5P-ThDP distortion and the unusually large bond length of the C2x-C3x scissile bond (1.61 ± 0.01 Å in the 4KXV high-resolution X-ray structure), which was proposed to be a consequence of the former, and to facilitate the progression over the following transition state, that involves the breaking of such abnormally long CC bond. Computer simulations are nowadays one of the best (and sometimes the only) way(s) to separate the individual physical components that contribute to the activation free energy and to quantify each of its contributions individually.

## 2 Methods

### 2.1 Enzyme Modeling

The molecular model used for the QM/MM calculations was obtained starting from the X-ray structure of hTK complexed with the ThDP-X5P adduct, at the resolution of 0.97 Å (PDB id: 4KXV), isolated from *Homo sapiens*.^20^ The crystal structure presents the active site at the homodimer interface, as canonically observed in other transketolases.^18,20−23^ Here, the substrate engages a hydrogen bond network with polarizable side chains of amino acid residues present in the catalytic task, as highlighted in Figure 1 and discussed in recent work.^19,20^

**Figure 2:**
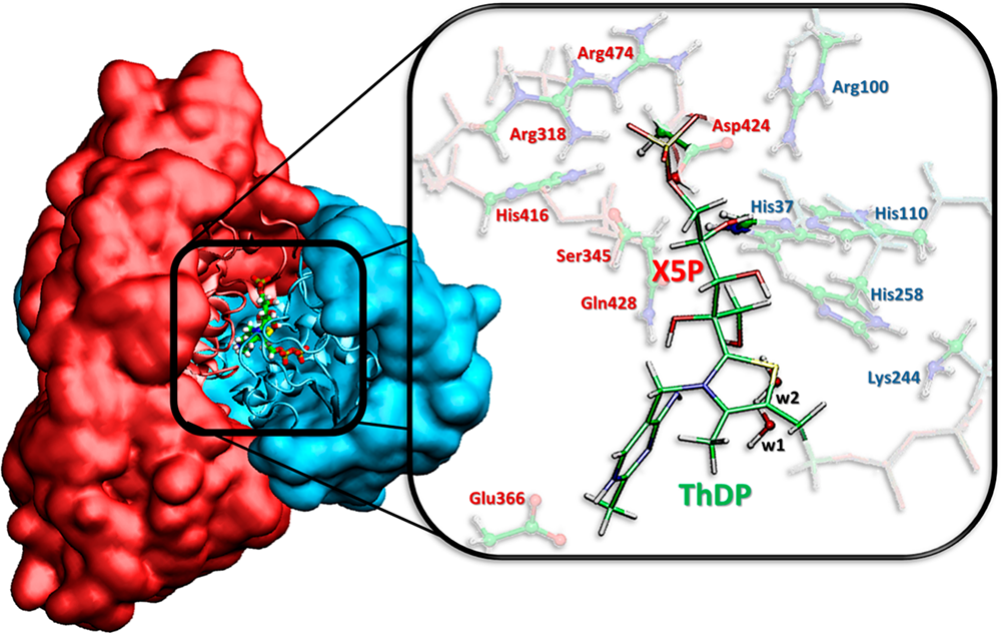
QM/MM model adopted starting from X-ray PDB code 4KXV. (Left) The entire protein, treated at the MM level of theory (chain A in cyan and chain B in red), and the DFT region are depicted on the left and on the right, respectively. (Right) Amino acid residues and water molecules are depicted in ball and stick while the adduct ThDP-X5P is represented in stick.

The complete protein complexed with ThDP-X5P has been inserted in a rectangular box (124 Å × 115 Å × 107 Å) of pre-equilibrated TIP3P water molecules^24^ and 4 Cl^–^ counterions to neutralize the total charge. The protonation states of ionizable residues were predicted by the H++ web server^25^ and further compared with the available experimental information (see Table S1 in the Supporting Information (SI)).^20,22,26^ Mutagenesis and kinetics studies on yeast transketolase suggested that the hTK dyad His258A-Lys244A may be involved in acid–base catalysis. According with this proposal, the Lys244A presents an uncommon, neutral state.^26^ As reported in Table S1, for Lys244A was calculated a p*K*_a_ value of 3.99, substantially lower than other values obtained for other residues protonated (p*K*_a_ > 10.00), in agreement with the proposal. Molecular mechanics parameters needed to be derived for the ThDP-X5P adduct. A geometry optimization at the HF/6-31G(d) level was performed and the restrained electrostatic potential^27^ (RESP) method was used to derive the ThDP-X5P atomic charges. The Antechamber tools, as implemented in the AMBER16 software package,^28^ were used to derive intramolecular parameters and Lennard-Jones parameters for ThDP-X5P, taken from the general Amber force field (GAFF).^29^ The file parameters are included in the SI, according to the AMBER force field format. The whole model was geometry-optimized with standard procedures.^30−32^ A progressive heating of 100 ps was performed from 0 to 310 K. A subsequent MD of 10 ns was carried out, in NPT condition at the temperature of 310 K and pressure of 1 bar, monitoring the conformational changes and the variations of relevant geometrical parameters involved in the catalytic mechanism (Figure S2). In all the simulations, the SHAKE^33^ algorithm, the PME^34^ scheme, and a cutoff radius of 12 Å were used. The QM/MM model was obtained applying the two-layers ONIOM formalism,^35^ starting from the minimized structure obtained at the *ff*99SB level of theory,^36^ and including in the high-level region (DFT) the residues His37^A^, Arg100^A^, His110^A^, Lys244^A^, His258^A^, Arg318^B^, Ser345^B^, Glu366^B^, His416^B^, Asp424^B^, Gln428^B^, and Arg474^B^ and two additional water molecules (w1 and w2), present in the crystal structure (Figure 2). w1 and w2 are involved in hydrogen bond networks with the O1–H group of the X5P substrate and protein surrounding, providing stable interactions in the active site. On the other hand, as further suggested by experimental evidence and demonstrated by previous theoretical work,^18−23^ the two water molecules do not play an active role in the proton transfers since the acid and basic groups were close enough (i.e., at hydrogen bond distance) to perform proton transfers without their assistance. In the low-level region (MM) was the remaining part of the protein, together with the 80 water molecules that were located within 5 Å from the active site cavity. The final model contains 19319 atoms. All atoms within a radius of 18 Å from the DFT region were geometry-optimized, while the water molecules and the remaining enzyme atoms were fixed in their initial positions.

### 2.2 ThDP-X5P Adduct Model and Its Analogues

The ThDP-X5P adduct intermediate present in the X-ray structure was extracted from the enzyme. All remaining atoms were deleted. This model was used to study the out-of-plane distortion present in the covalent intermediate without any influence from the enzyme scaffold.

**Figure 3:**
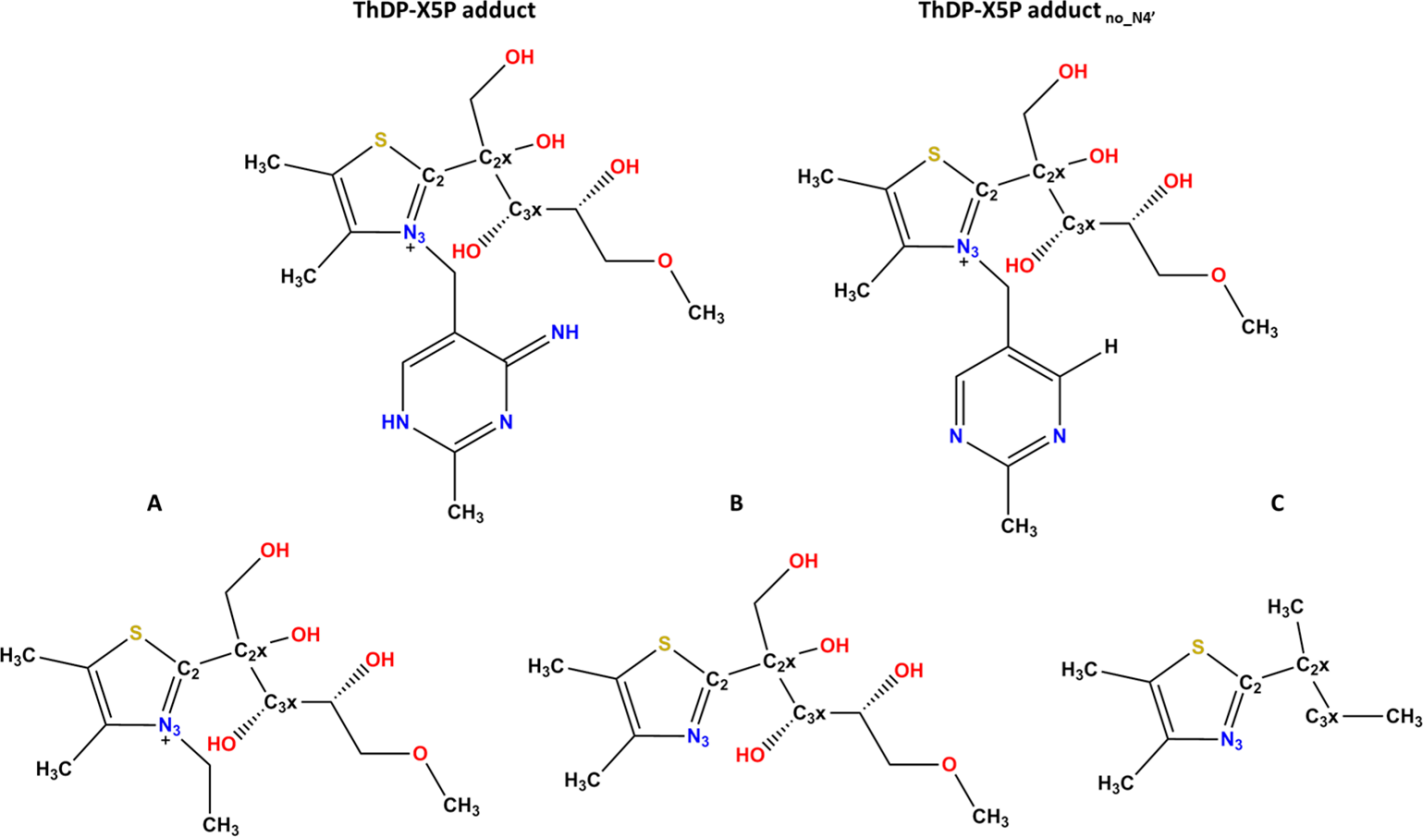
Small molecular models used to investigate the origin of the abnormal C2x–C3x bond length. Species **A** represents the ThDP-X5P adduct. Species **B** and **C** represent analogues of the same adduct but without the electron-withdrawing inductive/mesomeric/both effects on C2x and C3x.

We have replaced the −O–PO_3_^2–^ and −CH_2_–O–P_2_O_5_^2–^, of X5P and ThDP, respectively, with −OCH_3_ and −CH_3_ groups to facilitate comparison with results coming from a previous work.^16^ The final number of atoms was 61. Other smaller models were also used (species **A**–**C** and species ThDP-X5P_no_N4′_, Figure 3 and Figure S3), made just by deleting specific atoms of the ThDP-X5P adduct, as will be discussed in the main text. All these small models were studied with the same theoretical methods as the ThDP-X5P adduct model.

### 2.3 Technical Details

The QM/MM calculations were performed using the Gaussian09 software.^37^ The DFT region of the QM/MM system, as well as the whole ThDP-X5P adduct system and analogues, was described with the Becke exchange^38^ and Lee, Yang, and Parr^39^ correlation functionals. The 6-31G(d,p) basis set was used for geometry optimizations and the 6-311+G(2d,2p) basis set for single point energy calculations. In the QM/MM calculations, the *ff*99SB force field was used in the MM layer. The Coulomb interactions were evaluated using the electrostatic embedding scheme.^40^ The nature of each stationary point found along the potential energy surfaces (PESs) was confirmed by frequency calculations on the optimized structures at the same level of theory (Figure S4). The presence of a single imaginary vibrational frequency confirmed the nature of the transition state. Only vibrational temperatures larger than 100 K have been accounted during the calculation of entropy contribution, according with a validated procedure^41,42^ and successfully proposed in other works.^19,43,44^ The final free energy profiles were obtained adding the Zero Point Energy (ZPE), the empirical D3-dispersion correction,^45^ and rigid rotor/harmonic oscillator entropy contributions, as reported in Tables S2 and S3. Accurate energetic profiles were obtained through a relaxed scan along the dihedral angle involved in the distortion observed in the **INT1** (C5–S–C2–C2x). Dihedral angle values from 0° to 22° (with 1° increments) have been monitored and optimized. In order to improve the accuracy of the calculations, we performed single point calculations by using the aug-cc-pVDZ and aug-cc-pVTZ basis sets, and the aug-cc-pVDZ/C and aug-cc-pVTZ/C and the correlation fitting basis sets.^46^ These energies were used to extrapolate to the complete basis set (CBS) limit, according to Truhlar’s extrapolation scheme.^47^

In fact, the very high DLPNO-CCSD(T)/CBS level of theory was needed to reproduce the correct out-of-plane distortion. This makes it almost impossible to make QM/MM MD simulations with enough sampling using such a very high theoretical level. The contribution of entropy to a very small and local out-of-plane distortion is not expected to be relevant, and this is probably the reason why our methods match the experimental values so well. Therefore, the methodology is perfecty adequate for the problem under study.

Finally, the natural bond orbital (NBO) analysis has been performed on all stationary points.^48^

## 3 Results and Discussion

### 3.1 Formation of the X5P-ThDP Covalent Intermediate

The whole catalytic mechanism was studied in a previous work.^19^ Here we just focus on the first catalytic stage (two elementary reactions, Scheme 1), as the out-of-plane distortion takes place in this part of the catalytic cycle only. The ES → TS1 → **INT1** step describes the nucleophilic attack of the C2_ThDP_ carbanion on the C2x carbonyl carbon of X5P, producing the X5P-ThDP covalent adduct. We simulated this reaction step with the QM/MM model. The crucial aspect of this step is the C2–C2x bond formation. It starts from a value of 3.263 Å in the ES complex, achieving a value of 1.546 Å at **INT1**. A proton transfer from N4′_ThDP_ to O2_X5P_ (atom numbering in Scheme 1) also takes place. The C2–C2x bond is partially formed at TS1 but the N4′ proton is still not transferred, so the two connectivity changes are concerted but asynchronous. The free energy barrier to be overcome is 7.9 kcal·mol^–1^, and the reaction free energy was −7.0 kcal mol^–1^. Table S4 shows the NBO charges of relevant atoms for the catalytic process. The C2x-C3x scissile bond, which will be broken in the following step, has a length of 1.61 Å at **INT1**, which is significantly longer than typical single C–C bonds (usual bond length around 1.52 Å) and longer than all other substrate C–C bonds present in the QM layer. The bond length observed in the high-resolution 4KXV X-ray structure is also 1.61 ± 0.01 Å. The agreement between the experimental and computational bond lengths is excellent.

**Figure 4:**
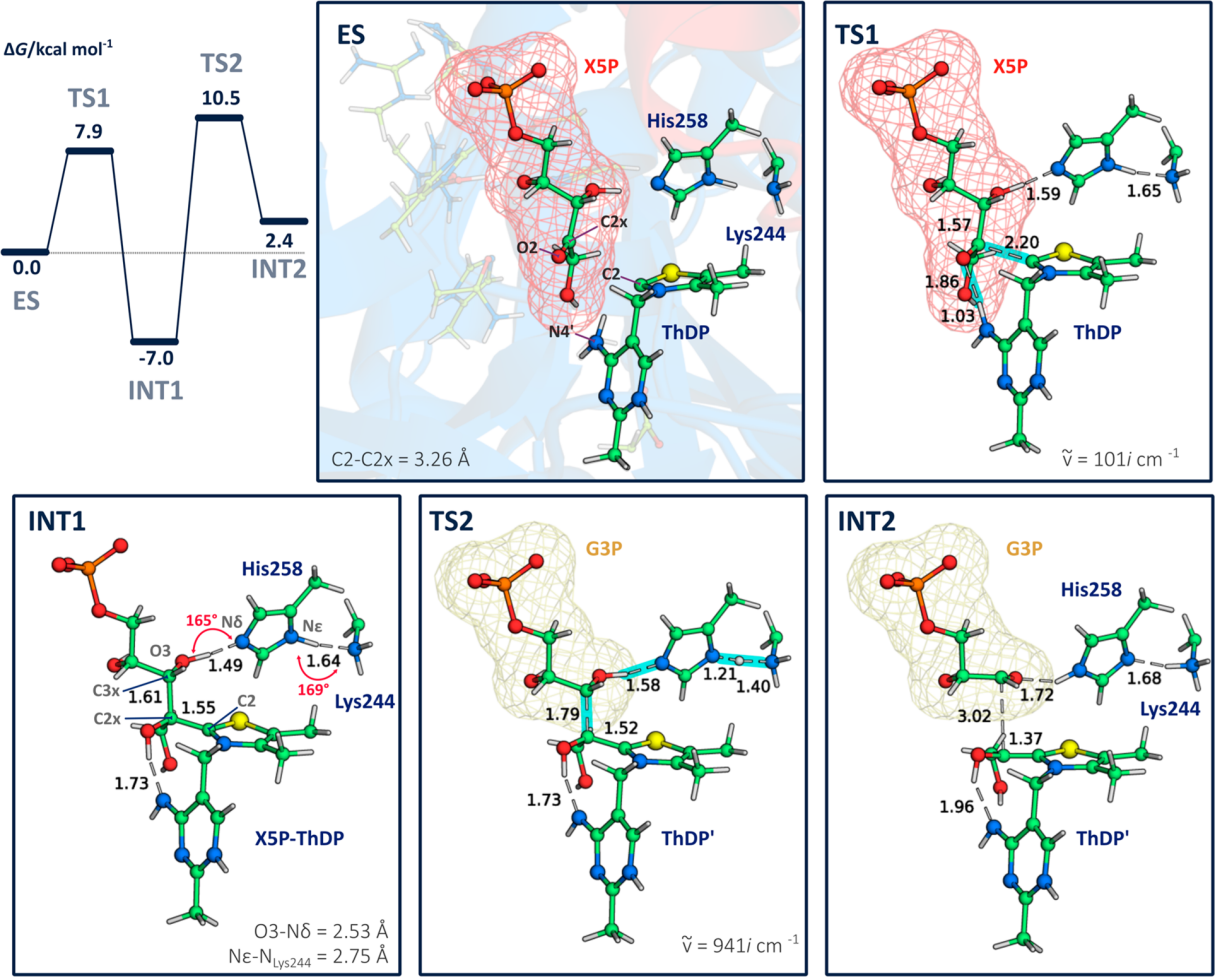
(Top left) Gibbs free energy profile for the conversion of X5P into G3P. **INT1** is the lowest free energy state of this stage, and very close to the absolute free energy minimum of the whole cycle (at 2.3 kcal·mol^–1^ from **INT4**, according to an earlier study^19^) that, according to the energy span model, makes the reaction rate to depend on this state. Therefore, preventing **INT1** from becoming too stable would have a catalytic effect in the overall reaction rate. Remaining panels: the geometry of the stationary states through the reaction of formation of the X5P-ThDP covalent intermediate and its transformation into G3P. Distances (Å) are reported in black and in red for bonds and geometrical parameters of hydrogen bond, respectively. The imaginary frequency values (cm^–1^) for TS1 and TS2 are reported.

The next step is the cleavage of the C2x–C3x bond, that generates the hTK_ThDP′_-G3P intermediate (INT2), marking the end of the first reaction stage. The latter reaction was simulated (Figure 4), and the free energy barrier for C2x–C3x bond cleavage amounted to 17.5 kcal·mol^–1^ and the reaction free energy of +2.4 kcal·mol^–1^. The reaction free energy for the whole stage is +2.4 kcal·mol^–1^. The overall free energy profile is similar in shape and in magnitude to the one found before using a cluster model.^19^ The difference of 6.9 kcal/mol between the **INT1**-TS2 barriers of the QM and QM/MM models can be accounted to the fact that moving from **INT1** to TS2 the large G3P portion moves significantly and such kind of rearrangements are differently described by the two models. A concerted asynchronous proton transfer, mediated by His258, also takes place in this step. The latter operates as the general base and acid, accepting a proton from the O3H group and delivering another one to Lys244 (Figure 4). The physically distorted **INT1** is the lowest energy species, and TS2 is the highest energy species of this stage,^1−6^ which makes them the rate-limiting species in this stage. If we look to the free energy profile of the whole cycle, calculated with a cluster model,^19^ we realize that TS2 is not rate-limiting but **INT1** is the second lowest free energy minimum, very close in energy to the absolute free energy minimum (difference of 2.3 kcal·mol^–1^). This means that if **INT1** is allowed to fully relax it can easily become the absolute minimum, thus raising the *k*_cat_ of the whole cycle. Therefore, the *intermediate destabilization* hypothesis, where the out-of-plane distortion would have the function of precluding **INT1** from becoming too stable, makes sense.^13^ The origin for the abnormal distance and weakened C2x–C3x bond was suggested to be also connected to this out-of-plane distortion.^20^ We will now analyze these two very relevant aspects in depth.

### 3.2 Out-of-Plane Distortion of the X5P-ThDP Intermediate

**Figure 5:**
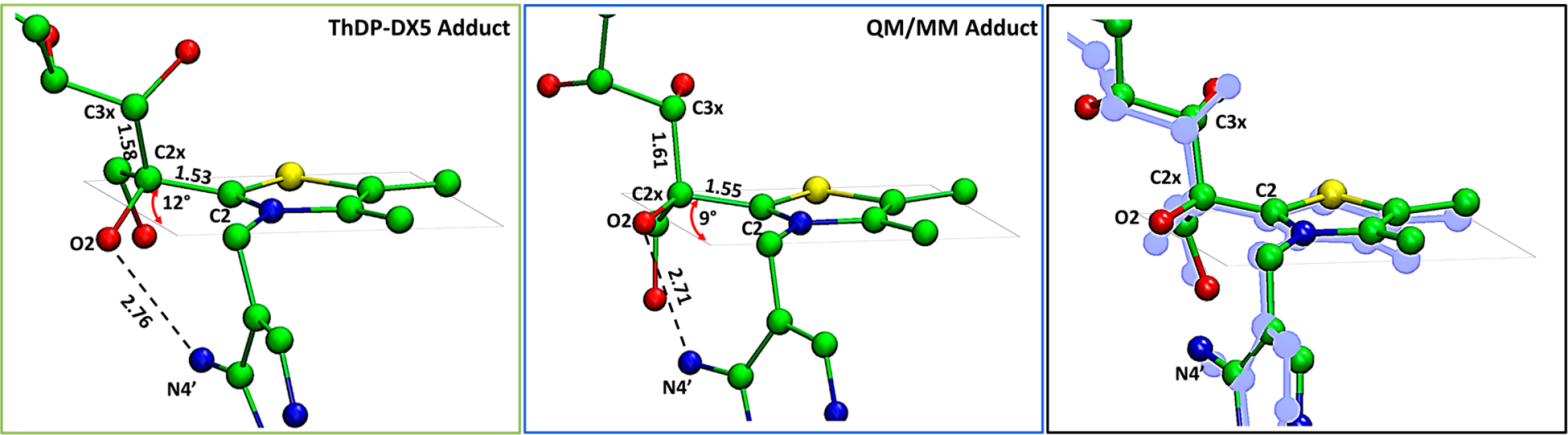
Out-of-plane distortion of the C2–C2x bond in the **two** used models. The models show a 9°–12° distortion when optimized at the B3LYP/6-31G(d). The ThDP-X5P system is free to rotate around the C2–C2x bond and avoid the repulsion between O2 and N4′ without clashing with the (absent) protein scaffold, eliminating the out-of-plane distortion, but the distorted geometry is still more favorable because it allows for the establishment of an internal O2–N4′ hydrogen bond. For clarity, residues retained during the QMMM calculations and hydrogen have not been shown. On the right, the superposition of the two models (in blue, the QMMM adduct).

To analyze and quantify the effect of the out-of-plane distortion of the C2–C2x bond in the free energy profile of the reaction, we have built an intentionally reduced molecular model (named ThDP-X5P adduct, Figure 5), consisting only in the X5P-ThDP adduct, stripped of the whole enzyme scaffold, to decouple the intrinsic energetics of the out-of-plane distortion from any other energy contribution emanating from the enzymatic scaffold (i.e., enzyme steric strain and interaction). Comparison of the results between this system and the whole enzyme QM/MM system will highlight the specific role to the protein scaffold on the distortion.

Upon free geometry optimization at the B3LYP/6-31G(d) level, the ThDP-X5P adduct displays an out-of-plane distortion of 12° (Figure 5). A deviation from the planarity of 9° is obtained by taking into account the effects of the neighboring residues in the QM/MM system. So, despite being stripped from the enzyme, the ThDP-X5P adduct shows a distortion that is similar to the one obtained in the enzymatic system. A previous study using a cluster model, treated at the same theoretical level as the QM layer here, arrived exactly to the same out-of-plane distortion (9°).^19^ The distortion in the X-ray structure is geometrically similar, but larger (22°). It is also significantly larger than the one found in other related ThDP enzymes that act on pyruvate, namely pyruvate oxidase and pyruvate dehydrogenase, where strain in the tetrahedral substrate-cofactor adducts led to 7–11° of distortion on the bond formed between the *sp*^*2*^ C2 of ThDP and the *sp*^*3*^ C2x relative to the planar aromatic thiazolium ring, as evidenced by the respective X-ray structures.^49,50^ In addition, the two systems studied here have the cofactor thiazolium and aminopyrimidine rings in a V-type arrangement, with an interatomic distance between the O2 and N4′ atoms of 2.75 and 2.71 Å. This matches very well with the V-type arrangement and the O2–N4′ value of 2.79 Å obtained in the hTK X-ray structure.^20^ This is an important fact because the short O2–N4′ distance has been pointed to as responsible for the out-of-plane distortion, as a planar structure would place the O2 and N4′ in a too close, repulsive contact.^20^

It may look surprising that the ThDP-X5P adduct, that is completely free to rotate around the C2x–C3x bond without clashing with the protein, avoiding in this way the bad contact between O2 and N4′, still shows an out-of-plane distortion without anything obvious forcing it. This fact indicates that the distortion is overall energetically favorable, probably because it allows the fine-tuning of the distance and angle of the O2–N4′ distance and permits the establishment of an O2–N4′ internal hydrogen bond (note that N4′ is protonated). Altogether, the establishment of a more favorable hydrogen bond seems to compensate for the distortion penalty. As such, the distortion energetic penalty has necessarily to be small.

**Figure 6:**
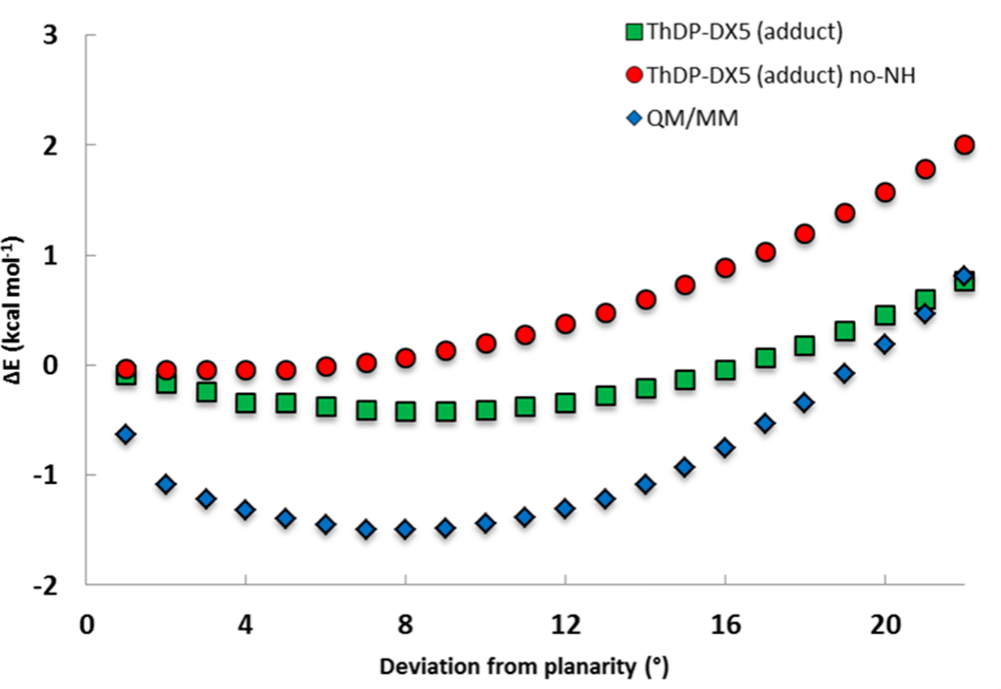
Energy profile as a function of out-of-plane distortion of the C2–C2x bond, from 0° to 22°, in the QM/MM model (blue), in the ThDP-X5P adduct (green), and in the same model but with the N4′ group replaced by hydrogen (red), which eliminates the putative clash and the internal hydrogen bond.

To measure the distortion penalty, and to check why the computational distortion (9°–12°) seems to deviate from the X-ray value (22°) we calculated a PES along the out-of-plane distortion angle, by rotating the S–C2 bond (Figure 6). Looking to the energy profile, we can see that the energy difference between the planar and the computational distorted geometries (9° and 12°) is very small (−1.5 kcal·mol^–1^ in the full enzyme model, −0.4 kcal·mol^–1^ in the ThDP-X5P system), as it is the difference in energy between the computational (9° and 12°) and the experimental distortion of 22° (less than +2.7 kcal·mol^–1^ when moving from the computational 9°–12° distortion to the experimental 22° distortion). The flatness of the PES is probably the reason why the experimental and computational distortions show differences of 11°–14°, and why the experimentally measured distortion changes by the same amount among similar enzymes;^51−55^ small differences in the medium (crystal vs solution), temperature, and/or small inaccuracies in the computations may easily translate in small energy differences, but the small energy differences translate into significant geometric differences due to the flatness of the PES, due to the high sensitivity of the geometry upon small energy changes. As such, a higher theoretical level might bring small changes to the energy but meaningful changes for the distortion angle. In this context, we recalculated the energy of a number of points of the PES of the ThDP-X5P system with the much higher and much more computational demanding DLPNO-CCSD(T)/CBS level (Table S5). Calculations at this very demanding level place the minimum in the PES precisely at 22°, in perfect agreement with experiments.

In summary, all computational results suggest that the distorted geometry is more stable than the planar geometry. However, as the energy difference between both geometries is rather small and lies close to the magnitude of the computational uncertainty, this conclusion has to be taken with care. What can safely be concluded is that the differences in energy between the two geometries (planar/distorted) are quite small, and in this way, the distortion will not have a significant effect on the overall reaction rate. Assuming an error bar of 1–2 kcal·mol^–1^ (a quite generous error bar for the relative energy of a small intramolecular distortion), the highest possible rate acceleration induced by the distortion would still fall below 1 order of magnitude (1.4 kcal·mol^–1^), which would be a meager contribution for the overall catalytic burden of the enzyme, considering that enzymes accelerate reaction rates in relation to the solution reaction by 10^6^–10^17^ fold.^56^ In summary, without denying the proposal that the ThDP-X5P out-of-plane distortion might translate into an *intermediate destabilization* catalytic effect, computer simulations predict that this effect, if existent, will be small. It is also interesting to note that if one considers a geometry with the experimental out-of-plane distortion value (22°) on the computational models (which is questionable as this 22° distortion does not lead to a computational stationary state) the effect of the out-of-plane distortion would be to lower the barrier for circa −1.2 kcal·mol^–1^. This effect, despite being catalytic, again reflects in an increase in the rate constant below 1 order of magnitude, which again is small in the context of typical enzyme proficiencies.

The role of the O2–N4′ hydrogen bond on the out-of-plane distortion has been evaluated, through the replacement of the N4′ group with a hydrogen atom in the ThDP-X5P adduct, generating the ThDP-X5P_no_N4′_ adduct model (incapable of establishing the internal hydrogen bond) and recalculating the PES along the rotation of the C2–C2x bond (Figure 6). The minimum energy structure is now a perfect planar configuration, without the earlier out-of-plane distortion, indicating that the favorable establishment of the internal O2–N4′ hydrogen bond is at the origin of the onset of an out-of-plane distortion. The ThDP-X5P_no_N4′_ molecular model allows decoupling of the energy penalty coming from the out-of-plane distortion from the stabilization energy coming from the internal O2–N4′ hydrogen bond. The out-of-plane distortion energy penalty corresponds to the energy difference between the structure of the ThDP-DX5_no_N4′_ model with the dihedral angle at 0° and at 12° (or at 0° and 22° if we want to use the experimental value for the distortion), and it corresponds to 0.2 and to 2.2 kcal·mol^–1^. To calculate the stabilizing effect of the internal hydrogen bond, we first calculate the difference between the energy of the ThDP-DX5 adduct at angle at 0° and at 12° (or between 0° and 22° if we want to use the experimental value for the distortion), which encompasses both the contributions of the out-of-plane distortion and hydrogen bonding, and then we subtract the out-of-plane distortion penalties calculated with the ThDP-DX5_no_N4′_ model. In this way, we obtain a value of −0.6 and −1.2 kcal·mol^–1^ for the stabilizing effect of the internal O2–N4′ hydrogen bond with a distortion of 12° and of 22°. A previous study on a similar ThDP-X5P adduct, at the DFT (B3LYP/6-31G(d)) level, indicated that forcing the planarity of the ThDP-X5P adduct without reorganizing its structure, and without forming the intramolecular 2-OH-N4′ hydrogen bond (due to the orientation of the hydrogen atoms that was specifically modeled) might involve a penalty of ∼20 kcal·mol^–1^.^16^ A full relaxation of the same model led to the establishment of an internal hydrogen bond between the 4-OH group of the substrate and the negative phosphate, making this more realistic, relaxed model, to lie in a different, much shallower, local minimum than the strained structure where this hydrogen bond was absent, a fact that precluded the evaluation of the real distortion energy.^16^ The fully relaxed model showed an out-of-plane deviation of 10°, in good agreement with the values found here. Finally, in the same study, a X5P-thiazolium model was geometry optimized and surprisingly displayed an out-of-plane distortion of 9°,^16^ despite the model lacking the N4’ amino group. This result is difficult to explain, and it is in contrast with our model where the N4′ amine was replaced by a hydrogen atom, which became perfectly planar upon geometry optimization.

### 3.3 The Very Elongated C2x–C3x Bond and Its Relationship with the X5P-ThDP Out-of-Plane Distortion

**Figure 7:**
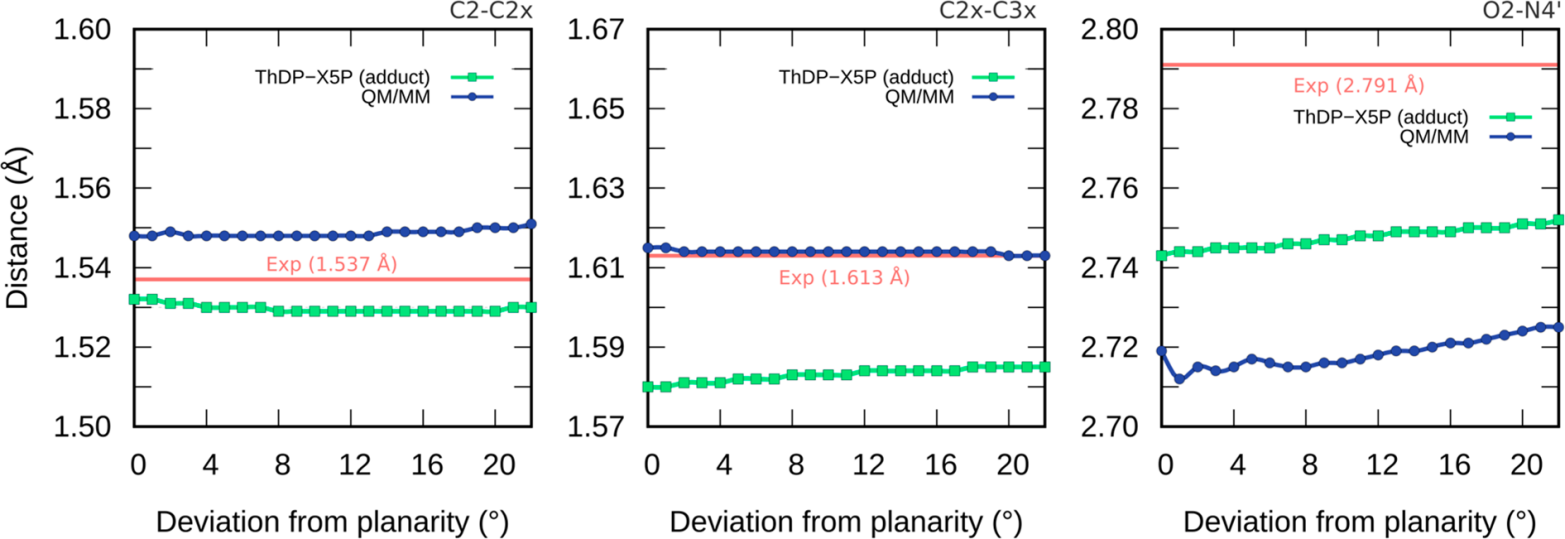
C2–C2x, C2x–C3x, and O2–N4’ distances as a function of the C5–S–C2-C2x dihedral, from 0° to 22° calculated at the B3LYP/6-31G(d) level.

We monitored the C2–C2x, C2x–C3x, and O2–N4′ distances as a function of the out-of-plane distortion (0°–22°). The results are shown in Figure 7.

The distances obtained by the relaxed scans are in excellent agreement with the experimental X-ray structure.^20^ Most importantly, there are no substantial changes in the three geometrical parameters, as the distortion grows from 0° to 22°. The C2x–C3x bond in particular is stretched in relation to a typical C–C single bond across the entire spectrum of out-of-plane distortions, including in the planar undistorted geometry. Therefore, the uncommon elongation of the C2x–C3x scissile bond seems not to be caused by the out-of-plane distortion of the ThDP-X5P adduct, contrarily to what has been previously thought. It looks probable that the C2x–C3x bond is unusually long due to the fact that both carbon atoms involved in the bond are significantly electron-deficient. C3x is electron deficient due to the electron-withdrawing inductive effect of the many OH groups and the phosphate group closely. C2x is electron-deficient due to the inductive effect of the hydroxyl groups and the mesomeric effect of the nitrogen atom bound to adjacent C2. The nitrogen has a propensity for withdrawing the π electrons from the C2=N3 double bond, leaving N3 less positive and C2 partially as a carbocation. To determine how much these effects contribute to the elongation of the scissile C2x–C3x bond, we modeled truncated derivatives of the ThDP-X5P adduct (**A**–**C**, Figure 3). Species **A** incorporates both the inductive and the mesomeric effects over the C2–C2x bond; species **B** still incorporates only the mesomeric effect but misses the inductive effects; species **C** incorporates none of the effects. We have optimized the geometry of the three species. The length of the C2–C2x bonds is respectively 1.60 Å, 1.58 Å, and 1.55 Å, following the expected tendency **A** > **B** > **C**. This indicates that the electron-deficiency of the C2 and C2x atoms due to the significant inductive/mesomeric effects that act upon them is at the origin of a significant part of the abnormal elongation of the C2–C2x. At the end the bond is still slightly elongated (the standard CC bond length is 1.52 Å), but most of the elongation can be explained by these inductive/mesomeric effects. The related optimized geometries are reported in Figure S3.

## 4 Conclusions

This work was devoted to investigating the concept of *intermediate destabilization* as an enzyme strategy to achieve catalysis. In the particular case studied here, we checked if an out-of-plane distortion of a key intermediate in the reaction cycle of hTK raised its energy and lowered the subsequent barrier, by stretching the scissile bond that will be broken in the subsequent reaction step. The computational results indicate that the out-of-plane distortion does not result from steric tension induced by the enzyme scaffold, as previously believed. Molecular models stripped from the enzyme, and free to relax, still show the out-of-plane distortion. The energy penalty associated with the distortion was found to be small (∼0.5 kcal·mol^–1^ at the computational minimum at 9°–12°) and the PES around the distortion to be very flat. The results suggest that the origin for the out-of-plane distortion is the establishment of an intramolecular hydrogen bond in a favorable geometry, whose stabilization energy (−0.7 to −1.2 kcal·mol^–1^ at 9°–12°) pays off the energy cost for distorting the planarity of the intermediate structure. We also suggest that the abnormally long length of the scissile C2x–C3x bond is not a consequence of the out-of-plane distortion, as previously believed, but mostly a product of significant electron-withdrawing effects on the carbon atoms making this bond. Thus far, the physical origin of the enzymatic efficiency of hTK is still a well-kept secret. Recent work^57^ has indicated that the preorganization of active sites may account for most of the catalytic effect.^11^ This may be a direction to understand the catalytic power of hTK.

## Supporting Information Available

Representation of human Transketolase (hTK); calculated p*K*_a_ for ionizable residues of hTK; RMSD of the hTK’s along of classical Molecular Dynamics (cMD). Relevant dihedral and distance variations in the X5P substrate, throughout of the cMD simulations; optimized geometries for small molecular models (A, B, and C species), at the B3LYP/6-31G(d) level of theory; energy contributions extrapolated for each stationary point (Method section); variation of charges, in NBO population analysis, of relevant atoms considered during the mechanism; single point energy at the DLPNO-CCSD(T)/CBS level of theory for the selected dihedral angles. The complete basis set (CBS) limit is calculated according to Truhlar’s extrapolation scheme (Results and Discussion section) (PDF)
ThDP-X5P adduct parameters (ZIP)
PDB coordinates of all stationary points of the reaction mechanism of TK (ZIP)
Animation for the reaction mechanism of X5P catalyzed by TK (MP4)
