# Supplementary material for: How the Destabilization of a Reaction Intermediate Affects Enzymatic Efficiency: The Case of Human Transketolase
Source: ACS Catal. 2020 Feb 7;10(4):2872–81. doi: 10.1021/acscatal.9b04690 (PMC8016368; doi:10.1021/acscatal.9b04690)
Supplement: Supplementary file 1 — cs9b04690_si_001.pdf [file cs9b04690_si_001.pdf]

Supporting Information:

How the Destabilization of a Reaction Intermediate Affects Enzymatic Efficiency: The Case of Human Transketolase

Mario Prejanò,[a‡] Fabiola E. Medina,[b‡] Maria J. Ramos,[b] Nino Russo,[a] Pedro A. Fernandes,[b*] Tiziana Marino[a*]

[a]*Dipartimento di Chimica e Tecnologie Chimiche, Università della Calabria, 87036 Arcavacata di Rende (CS) (Italy)*

[b]*UCIBIO, REQUIMTE, Departamento de Química e Bioquímica, Faculdade de Ciências, Universidade do Porto, Rua do Campo Alegre s/n, 4169-007 Porto, Portugal*

[‡]These two authors equally contributed.

Author Information

Corresponding Authors

[a]*E-mail for T.M: tiziana.marino65@unical.it

[b]*E-mail for P.A.F: pafernan@fc.up.pt

INDEX

1. METHODS SECTION ______ S3

Figure S1. (left) Cartoon representation of human Transketolase (hTK). **(right)** Residues that interact with the X5P substrate (dark blue, ball-and-stick). Catalytic residues are colored in cyan (chain A) and red (chain B). S3

Table S1. Calculated p*Ka* for ionizable residues of hTK. Residues fully protonated (positively charged) or deprotonated (negatively charged) are highlighted in blue and red, respectively. The QM residues are in green. ______ S4

Figure S2. a) Root Mean Square Deviation (RMSD) of the hTK's along of classical Molecular Dynamics (10 ns). **b)** (top) Relevant dihedral and (bottom) distance variations in the X5P substrate, throughout of the cMD simulations. ______ S5

Figure S3. Optimized geometries for A, B and C species, at B3LYP/6-31G(d) level of theory. In order to simplify the figure, the hydrogens are not reported. ______ S5

Table S2. Energy contributions extrapolated for each stationary points. All values are in kcal·mol^{-1}. ______ S6

Table S3. Energy contributions extrapolated by PES for each stationary point. ______ S6

2. RESULTS SECTION ______ S6

Table S4. Variation of charges, in NBO population analysis, of relevant atoms considered during the mechanism. The charges are in a.u. ______ S6

Table S5. Single point energy (kcal·mol^{-1}) at DLPNO-CCSD(T)/CBS level of theory for the selected dihedral angles. The complete basis set (CBS) limit is calculated according to Truhlar's extrapolation scheme (1). The highlighted (red) column represents the minimum in the PES. ______ S7

REFERENCE ______ S7

ADDITIONAL SUPPORTING INFORMATION FILES

ThDP-X5P adduct parameters

PDB coordinates of all stationary points of the reaction mechanism of TK

Animation for the reaction mechanism of X5P catalyzed by TK

1. METHODS SECTION

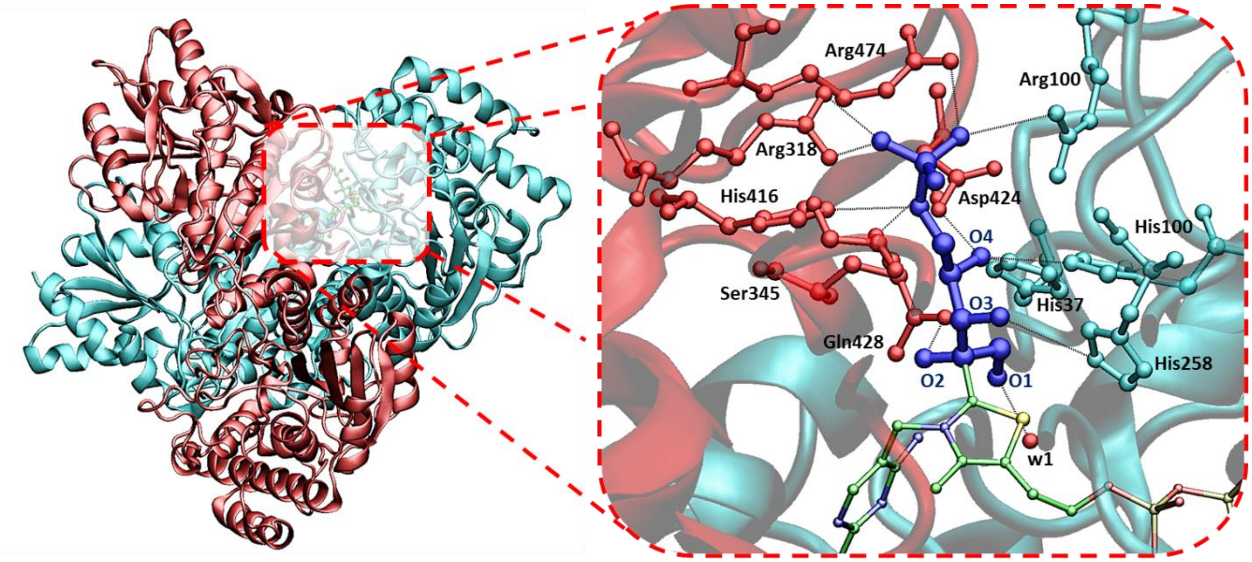

Figure S1. (left) Cartoon representation of human Transketolase (hTK). **(right)** Residues that interact with the X5P substrate (dark blue, ball-and-stick). Catalytic residues are colored in cyan (chain A) and red (chain B).

Table S1. Calculated p*Ka* for ionizable residues of hTK. Residues fully protonated (positively charged) or deprotonated (negatively charged) are highlighted in blue and red, respectively. The QM residues are in green.

Residue	pK_a	Residue	pK_a	Residue	pK_a	Residue	pK_a
TYR-4	>12.000	TYR-173	>12.000	LYS-327	>12.000	LYS-499	10.336
HIS-5	6.015	LYS-174	10.689	HIS-330	8.293	ASP-500	3.226
LYS-6	10.97	ASP-176	0.969	ASP-333	2.754	ASP-501	<0.000
ASP-8	1.944	ASP-183	<0.000	ARG-334	>12.000	HIS-513	7.023
LYS-11	9.886	ARG-186	>12.000	ASP-339	<0.000	GLU-514	<0.000
LYS-16	10.293	ASP-191	3.209	ASP-341	4.308	GLU-520	3.849
ASP-17	0.229	HIS-197	5.179	LYS-343	>12.000	LYS-523	10.764
ARG-21	>12.000	ASP-200	3.698	GLU-349	3.158	LYS-524	10.581
ARG-23	>12.000	TYR-202	>12.000	LYS-352	9.784	GLU-525	1.816
**HID-37**	<0.000	LYS-204	10.072	LYS-353	11.124	LYS-526	10.777
CYS-41	>12.000	ARG-205	>12.000	GLU-354	2.149	ARG-530	>12.000
CYS-42	>12.000	CYS-206	>12.000	HIS-355	4.372	ASP-533	<0.000
GLU-46	<0.000	GLU-207	4.249	ASP-357	2.687	LYS-538	>12.000
HIS-54	<0.000	HIS-212	3.755	ARG-358	>12.000	ASP-541	1.472
ARG-57	>12.000	ASP-217	1.926	GLU-361	1.692	ARG-542	>12.000
TYR-58	>12.000	HID-219	<0.000	CYS-362	>12.000	LYS-543	>12.000
LYS-59	11.664	GLU-222	3.587	TYR-363	>12.000	ASP-547	2.61
ASP-62	1.837	GLU-223	3.26	**GLU-366***	4.854	ARG-550	>12.000
ARG-64	>12.000	CYS-225	>12.000	CYS-376	>12.000	LYS-553	10.847
HIS-67	<0.000	LYS-226	10.557	ARG-379	>12.000	ARG-555	>12.000
ASP-69	<0.000	LYS-232	10.932	ARG-381	>12.000	GLU-560	<0.000
ARG-70	>12.000	HIS-233	5.946	CYS-386	>12.000	ASP-561	<0.000
LYS-75	>12.000	LYS-241	11.434	ARG-395	>12.000	HID-562	<0.000
HIS-77	<0.000	**LYS-244**	3.989	ASP-398	<0.000	TYR-563	>12.000
TYR-83	>12.000	ARG-246	>12.000	ARG-401	>12.000	TYR-564	>12.000
GLU-88	<0.000	GLU-252	0.619	GLU-407	4.557	GLU-565	4.777
GLU-94	4.344	ASP-253	<0.000	CYS-413	>12.000	GLU-570	2.562
GLU-96	2.691	LYS-254	11.274	**HIS-416**	<0.000	GLU-579	2.89
**ARG-100***	11.014	GLU-255	3.722	CYS-417	>12.000	HIS-586	10.084
LYS-102	11.102	**HIS-258**	<0.000	GLU-423	4.917	ARG-591	>12.000
ASP-106	1.886	LYS-260	10.31	**ASP-424**	10.17	ARG-594	>12.000
ASP-108	0.313	LYS-264	9.785	GLU-432	5.576	LYS-597	10.837
HID-110	2.588	GLU-268	4.244	ASP-433	<0.000	GLU-600	3.17
LYS-114	>12.000	GLU-273	3.795	ARG-438	>12.000	LYS-603	11.053
ASP-119	<0.000	TYR-275	9.987	TYR-447	>12.000	ASP-608	3.924
CYS-133	>12.000	LYS-281	>12.000	ASP-450	<0.000	ARG-609	>12.000
TYR-137	>12.000	LYS-282	10.212	GLU-455	<0.000	ASP-610	3.639
LYS-140	>12.000	LYS-283	>12.000	LYS-456	>12.000	ARG-617	>12.000
TYR-141	>12.000	GLU-291	2.675	GLU-459	2.998		
ASP-143	<0.000	ASP-292	4.808	LYS-465	10.896		
LYS-144	11.549	ASP-297	4.127	CYS-468	>12.000		
TYR-147	>12.000	ARG-302	>12.000	ARG-471	>12.000		
ARG-148	>12.000	TYR-309	>12.000	ARG-474	>12.000		
TYR-150	>12.000	LYS-310	>12.000	GLU-476	1.999		
CYS-151	>12.000	ASP-313	2.32	TYR-481	>12.000		
ASP-155	9.013	LYS-314	9.516	GLU-485	2.994		
GLU-157	<0.000	**ARG-318***	>12.000	ASP-486	5.074		
GLU-160	4.783	LYS-319	9.679	LYS-493	>12.000		
GLU-165	4.327	TYR-321	>12.000	LYS-497	10.04		

*****QM residues.

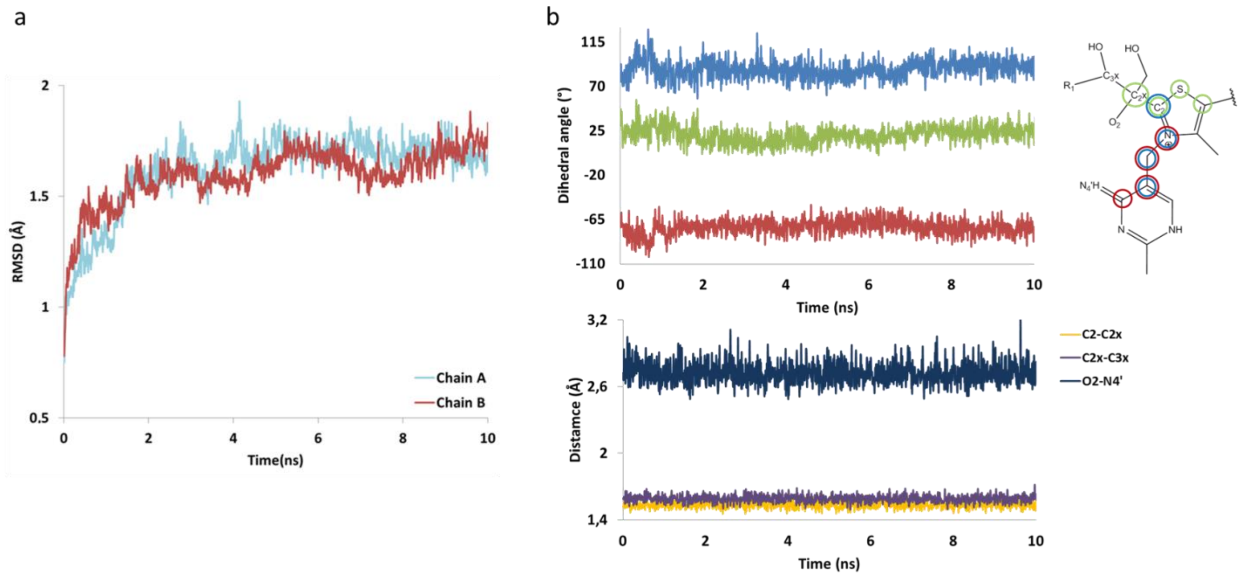

Figure S2. a) Root Mean Square Deviation (RMSD) of the hTK's along of classical Molecular Dynamics (10 ns). **b)** (top) Relevant dihedral and (bottom) distance variations in the X5P substrate, throughout of the cMD simulations.

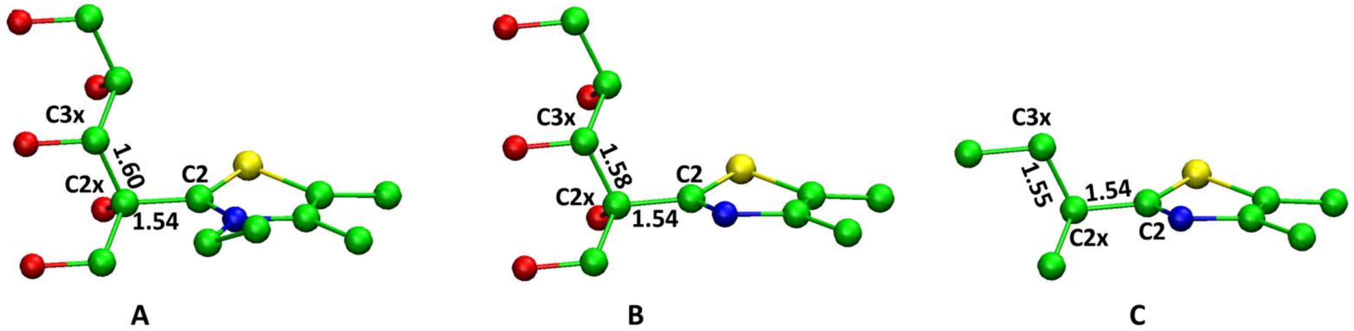

Figure S3. Optimized geometries for A, B and C species, at B3LYP/6-31G(d) level of theory. In order to simplify the figure, the hydrogens are not reported.

Table S2. Energy contributions extrapolated for each stationary points. All values are in kcal·mol^{-1}.

	Δ $E_{B3LYP-D3}$	Δ ZPE	-TΔS	ΔG
**ES**	0.0	0.0	0.0	0.0
**TS1**	9.4	0.4	-1.9	7.9
**INT1**	-7.1	1.6	-1.5	-7.0
**TS2**	15.3	-3.5	-1.3	10.5
**INT2**	2.5	1.4	-1.5	2.4

Table S3. Energy contributions extrapolated by PES for each stationary point.

Species	E_{B3LYP} (a.u.)	E_{ZPE} (a.u.)	E_{D3} (a.u.)	E_{total} (a.u)
**ES**	-5066.062893	77.194208	-0.222180	-4990.738904
**TS1**	-5066.044235	77.194826	-0.230321	-4990.723260
**INT1**	-5066.068333	77.196703	-0.228614	-4990.747778
**TS2**	-5066.042222	77.188604	-0.228851	-4990.720104
**INT2**	-5066.050986	77.196389	-0.227163	-4990.732741

2. RESULTS SECTION

Table S4. Variation of charges, in NBO population analysis, of relevant atoms considered during the mechanism. The charges are in a.u.

	C2	C2x	O2	C3x	O3
**ES**	-0.26	0.57	-0.57	0.00	-0.83
**TS1**	-0.13	0.49	-0.71	0.03	-0.83
**INT1**	0.12	0.21	-0.79	0.09	-0.83
**TS2**	0.12	0.21	-0.79	0.13	-0.82
**INT2**	0.00	0.21	-0.78	0.33	-0.68

Table S5. Single point energy (kcal·mol^{-1}) at DLPNO-CCSD(T)/CBS level of theory for the selected dihedral angles. The complete basis set (CBS) limit is calculated according to Truhlar's extrapolation scheme (1). The highlighted (red) column represents the minimum in the PES.

Dihedral angle (^{0})	Reference energy (1)	Reference energy (2)	Correlation energy (3)	Correlation energy (4)	ΔE (Ref. energy)	ΔE (Corr. energy)	‡ΔE_{total}
	**aug-cc-pVDZ aug-cc-pVDZ/C** (a.u.)	**aug-cc-pVTZ aug-cc-pVTZ/C** (a.u.)	**aug-cc-pVDZ aug-cc-pVDZ/C** (a.u.)	**aug-cc-pVTZ aug-cc-pVTZ/C** (a.u.)	**CBS** (kcal·mol^{-1})	**CBS** (kcal·mol^{-1})	**DLPNO-CCSD(T)/CBS** (kcal·mol^{-1})
**0**	-2174.960	-2175.350	-5.180	-6.158	0.000	0.000	0.00
**9**	-2174.961	-2175.350	-5.180	-6.158	0.122	-0.062	0.06
**15**	-2174.961	-2175.350	-5.181	-6.158	0.049	-0.539	-0.49
**18**	-2174.960	-2175.350	-5.181	-6.159	-0.021	-0.776	-0.80
**22**	-2174.959	-2175.350	-5.182	-6.160	-0.179	-1.124	-1.30
**25**	-2174.958	-2175.348	-5.181	-6.160	1.166	-2.126	-0.96
**28**	-2174.956	-2175.347	-5.183	-6.161	2.115	-1.812	0.30

‡E_{tot} (CBS) = C1*(2) - C2*(1) + C3*(4) - C4*(3); coefficients: C1 and C2 (1.199905), C3 (1.584336), and C4 (0.584336).

REFERENCE

1. Truhlar, D. G. Basis-Set Extrapolation. *Chem. Phys. Lett.* **1998**, *294*, 45-48.
